# Regulating a key mitotic regulator, polo‐like kinase 1 (PLK1)

**DOI:** 10.1002/cm.21504

**Published:** 2018-12-07

**Authors:** Erica G. Colicino, Heidi Hehnly

**Affiliations:** ^1^ Department of Cell and Developmental Biology Upstate Medical University Syracuse New York; ^2^ Department of Biology Syracuse University Syracuse New York

**Keywords:** biosensor, cell division, centrosome, chemical genetics, FRET, kinetochore, midbody, mitosis, polo‐like kinase 1, scaffolds

## Abstract

During cell division, duplicated genetic material is separated into two distinct daughter cells. This process is essential for initial tissue formation during development and to maintain tissue integrity throughout an organism's lifetime. To ensure the efficacy and efficiency of this process, the cell employs a variety of regulatory and signaling proteins that function as mitotic regulators and checkpoint proteins. One vital mitotic regulator is polo‐like kinase 1 (PLK1), a highly conserved member of the polo‐like kinase family. Unique from its paralogues, it functions specifically during mitosis as a regulator of cell division. PLK1 is spatially and temporally enriched at three distinct subcellular locales; the mitotic centrosomes, kinetochores, and the cytokinetic midbody. These localization patterns allow PLK1 to phosphorylate specific downstream targets to regulate mitosis. In this review, we will explore how polo‐like kinases were originally discovered and diverged into the five paralogues (PLK1‐5) in mammals. We will then focus specifically on the most conserved, PLK1, where we will discuss what is known about how its activity is modulated, its role during the cell cycle, and new, innovative tools that have been developed to examine its function and interactions in cells.

## 1 INTRODUCTION

Mitosis incorporates a multitude of protein interactions and macromolecular machinery to successfully segregate sister chromatids into new daughter cells (Nigg & Stearns, 2011). The fidelity of mitotic progression requires regulatory and signaling proteins to be dynamically recruited and constrained at centrosomes. The centrosome is a structure that is composed of two centrioles that start their duplication cycle during S‐phase (reviewed in Nigg & Stearns, 2011). Once the centrosome has duplicated into two mitotic centrosomes, they will then drive the assembly of the microtubule‐based spindle. The assembly of the microtubule‐based spindle requires the recruitment of microtubule nucleating components (e.g., pericentrin, γ‐tubulin, CEP215, γ‐turc, CEP170, CEP68, AKAP450, etc.) (Choi, Liu, Sze, Dai, & Qi, 2010; Doxsey, Steln, Evans, Calarco, & Kirschnefi, 1994; Fabbro et al., 2005; Kolobova et al., 2017; Zimmerman, Sillibourne, Rosa, & Doxsey, 2004). The recruitment of these components require activated mitotic signaling cascades involving the mitotic kinases Aurora A, polo‐like kinases (PLKs), and cyclin‐dependent kinase 1 (CDK1) to name a few (Bruinsma et al., 2015; Hehnly et al., 2015; Lee et al., 2018; Sanhaji et al., 2014; Thomas et al., 2016). One kinase that seems to be at the center of this process from a single cell eukaryote to a multi‐cellular vertebrate is polo‐like kinase 1 (PLK1).

PLK, in mammals, has diverged into five paralogues, PLK1‐5 (De Cárcer, Manning, & Malumbres, 2011). Unlike its alternative mammalian paralogues (PLK2‐5), PLK1 function is evolutionarily conserved persisting in *Schizosaccharomyces pombe* (fission yeast), *Drosophila melanogaster* (fruit flies), and *Caenorhabditis elegans* (PLK‐1) where it acts broadly throughout mitosis from G2 until the final stage of cytokinesis, abscission (Nasmyth & Nurse, 1981; Sunkel & Glover, 1988). While these initial studies defined an essential and conserved role for PLK1 during the cell cycle, it has been difficult to delineate the spatial and temporal regulation of PLK1. However, with the onset of chemical genetics and biosensors to analyze PLK1 activity in live cells, giant strides have been made, and likely will still be made, in understanding PLK1 activity and function during the cell cycle (Bruinsma et al., 2014, 2015; Burkard et al., 2007; Lera & Burkard, 2012; Liu, Davydenko, & Lampson, 2012; Macůrek et al., 2008). In this review, we will touch briefly on how polo‐like kinases were discovered, as well as its evolutionary conservation and divergence in mammals. We will then specifically focus on PLK1, where we will explore its diverse functions, subcellular localizations, scaffold–protein interactions, and its known downstream phosphorylation substrates. Finally, we will discuss the intramolecular tools that will facilitate future advancements in understanding PLK1’s role throughout the cell cycle.

## 2 DISCOVERY OF POLO‐LIKE KINASES

Polo‐like kinase was first identified in budding yeast (*Saccharomyces cerevisiae*) through the screening of various cell division cycle (cdc) mutants (Hartwell, Mortimer, L O, CuLo, & Esposito, 1973). Hartwell et al. defined and characterized previously identified temperature‐sensitive mutants, which perturbed the progression of the cell cycle. They determined that *cdc5*, a homologue of mammalian PLK1, specifically caused cytokinetic defects when cells were shifted to a restrictive temperature, resulting in a significant increase in bi‐nucleated cells. Strikingly, if the cells were shifted to restrictive temperatures following cytokinesis completion, cells would then arrest during the second cell cycle. This work demonstrated that the temporal regulation of cdc5/PLK1 is necessary for the initiation and proper progression of mitosis (Hartwell et al., 1973). PLK1’s role in mitotic progression was later confirmed in *Drosophila melanogaster* (Sunkel & Glover, 1988). In *Drosophila*, the homologue is called *polo*. Homozygous *polo* mutant flies were found to arrest in development, failing to form a fully developed embryo. This was due to cells of *polo* embryos arresting at the same stage of the cell cycle, prometaphase and metaphase, where they presented with multipolar spindles, aneuploidy, and abnormal centrosome structure and microtubule aster formation (Sunkel & Glover, 1988). This suggested that the gene *polo* was a vital mitotic regulator that assists in the proper establishment of the mitotic spindle through microtubule nucleation from the mitotic centrosomes. When this process is disrupted, a functional bipolar spindle cannot be formed and chromosome mis‐segregation occurs, resulting in increased aneuploidy (Sunkel & Glover, 1988).

While it was becoming clear that PLK1 is a vital regulator of mitosis, scientists were unclear as to ***how*** PLK1 regulated this process. By further examining the *Drosophila polo* sequence, it was found that *polo* contains an N‐terminal sequence consistent with serine–threonine kinases such as SNF1, KIN1, and KIN2 in budding yeast (Llamazares et al., 1991). It was later discovered through indirect protein kinase analysis of *polo* from mitotic *Drosophila* lysates, that PLK1 was capable of phosphorylating the protein casein, further supporting the role of PLK1 as a kinase (Fenton & Glover, 1993). A later study confirmed that this PLK1 kinase activity was specific during mitosis by performing in vitro kinase assays with cells released into prometaphase after nocodazole treatment. Casein phosphorylation by PLK1 was specific to lysates for 50 min after nocodazole release (Lee, Yuan, Kuriyama, & Erikson, 1995). Over time, it was found that this kinase was highly conserved between vertebrates, invertebrates, and single‐celled organisms alike. This includes *Caenorhabditis elegans* PLK‐1 (Ouyanga, Wangb, & Daia, 1999), *Xenopus* (*Plx1*) (Descombes & Nigg, 1998), and mammals (*PLK1*) (Lee & Erikson, 1997). The evolutionary conservation of this gene and role as a mitotic kinase strengthened the notion that this is a vital component of cellular division.

## 3 FUNCTIONS OF THE DIVERGENT MAMMALIAN PLKS (1–5)

**Figure 1:**
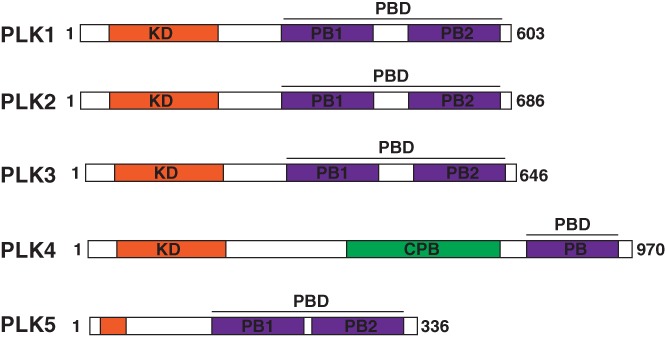
There are five mammalian polo‐like kinase paralogues. The five mammalian paralogues contain an N‐terminal catalytic kinase domain (orange) and a C‐terminal polo‐box domain (PBD, purple). The PLK4 cryptic polo‐box domain (CPB) is shown in green. Adapted from (De Cárcer, Manning, et al., 2011)

Mammals contain five paralogues (PLK1‐5) that are distinct in localization, expression patterns, and function within cells. Despite this divergence, each of the paralogues retained the canonical PLK domains, the N‐terminal kinase domain and the C‐terminal polo‐box domain (PBD) (depicted in Figure 1). The kinase domain is a catalytic T‐loop domain which allows PLKs to convert ATP to ADP, transferring the phosphate group to any of the numerous PLK downstream phosphorylation targets (Kothe et al., 2007). The PBD domain is unique in that it recognizes specific phosphorylation motifs on PLK binding scaffolds (Elia, Rellos, et al., 2003; Elia, Cantley, & Yaffe, 2003). The main difference between the five PLK paralogues is the number of PBDs they contain, their expression patterns within particular cell types, and/or their expression patterns throughout the cell cycle. For instance, PLK4 has diverged to contain a cryptic polo‐box domain (CPB) and a single polo‐box region within the PBD overtime (Figure 1; De Cárcer, Escobar, et al., 2011; Habedanck, Stierhof, Wilkinson, & Nigg, 2005). Due to the presence of the cryptic polo‐box and single PBD, PLK4 homodimerizes, altering its structural conformation and causing regulated spatial activity in cells (Leung et al., 2002; Sillibourne & Bornens, 2010). This localizes the kinase to the centrosome during S‐phase to bind scaffolds, such as CEP192 and CEP152 (Park et al., 2014; Slevin et al., 2012; Sonnen, Gabryjonczyk, Anselm, Stierhof, & Nigg, 2013), which restricts its localization and activity to the centrosome allowing for precise spatial regulation of centrosome duplication (Leung et al., 2002; Sillibourne & Bornens, 2010; Sonnen et al., 2013). This led researchers to further try and understand how either a specific PBD or the number of polo‐box regions leads to scaffold‐binding specificity and PLK localization patterns. To answer this question, PLK2‐4 chimeras containing PLK1 kinase domains were expressed in cells depleted of endogenous PLK1. These chimeric PLKs were able to rescue phenotypes that are present with PLK1‐depletion (Van De Weerdt et al., 2008), supporting the idea that, through the PBDs, specific scaffolding of polo‐kinases is required for PLK spatial localization and temporal phosphorylation of downstream phosphorylation targets. A schematic representation of the five PLKs containing both their kinase and PBDs are demonstrated in Figure 1. The PBD is highly conserved between PLK1‐3 (36–43% homology) and is less so in PLK4 (16% homology) (Park et al., 2010). Thus, this brings up the question, what is it about these different PLKs that allow directed localization and subsequent downstream function?

One possibility is that the expression patterns within a cell and within an organism varies between PLKs. PLK2, PLK3, and PLK5 are expressed predominantly during interphase and play a variety of functions. PLK2 has been implicated in neuronal synapse function and signal transduction (Seeburg, Pak, & Sheng, 2005). Additionally, PLK2 has been implicated during S‐phase of the cell cycle, phosphorylating the protein CPAP and allowing for subsequent centriole assembly (Chang, Cizmecioglu, Hoffmann, & Rhee, 2010). PLK5 is unique in humans in that it has lost the kinase domain, but retained the conserved PBD‐domain characteristic of the PLK‐family kinases, gaining its membership into this family (Andrysik et al., 2010; De Cárcer, Escobar, et al., 2011). Like PLK2, PLK5 functions exclusively within quiescent brain cells where it aids in the formation of neuritic processes (De Cárcer, Escobar, et al., 2011). Even less is currently known about PLK3, but studies have implicated it during G0/G1, during apoptosis, and later during S/G2‐phase as a component of p53‐dependent DNA damage checkpoint (Zimmerman & Erikson, 2007). PLK1 and PLK4 are expressed predominantly during S/G2 and M‐phase of diving cells.

**Figure 2:**
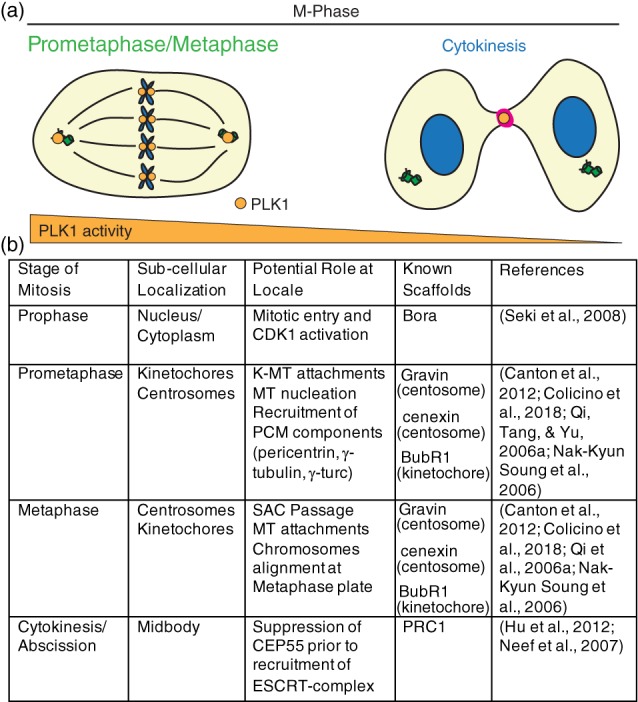
PLK1 subcellular distribution and function during metaphase in mammalian cells. (a) PLK1 (orange) localizes during M‐phase to the mitotic centrosomes, kinetochores, and cytokinetic midbody (magenta) to ensure mitotic progression, microtubule attachments, and anaphase onset, as well as proper cytokinesis and abscission. Gradient below (orange) represents relative PLK1 activity changes between prometaphase/metaphase and cytokinesis. (b) Table outlining PLK1 localization patterns with corresponding functions and known binding scaffolds during M‐phase

PLK1 and PLK4 are the most heavily studied PLKs. PLK1 is the most highly conserved through single celled eukaryotes up to vertebrates where it is expressed during the G2/M‐phases of the cell cycle (Figure 2a). This expression is the result of p53‐dependent transcription which follows the upregulation of two key factors during division, CDK1 and Cyclin B (Martin & Strebhardt, 2006). These proteins work in concert with PLK1 to ensure proper mitotic progression. CDK1/Cyclin B phosphorylates specific serine/threonine residues on PLK1‐binding scaffolds, with the sequence motif of Ser‐pSer/pThr‐Pro/X, allowing PLK1 to directly bind. Known PLK1 scaffold proteins include Bora (Seki, Coppinger, Jang, Yates, & Fang, 2008), cenexin (Soung et al., 2006), Gravin (Canton et al., 2012), BubR1 (Qi, Tang, & Yu, 2006), PRC1 (Hu, Özlü, Coughlin, Steen, & Mitchison, 2012) to name a few, and localize subcellularly to the nucleus, centriole appendages, pericentriolar matrix (PCM), kinetochores, and cytokinetic midbody, respectively (Figure 2b). These scaffolds aid PLK1 in its regulation of centrosome maturation and microtubule nucleation, as well as regulating its role as a component of the spindle assembly checkpoint, during which it ensures proper end‐on microtubule attachments at kinetochores and activation of the anaphase promoting complex (APC). The APC is an E3‐ubiquitin ligase that degrades mitotic proteins, allowing sister chromatids to separate and cells to progress through the end of mitosis and enter G1 (Eckerdt & Strebhardt, 2006). Since PLK1 regulates the onset of this complex, PLK1 is known as a major regulator of the metaphase to anaphase transition of division.

PLK4 is exclusively expressed during S‐phase of the cell cycle. The expression and localization of PLK4 at the centrioles triggers centriole duplication and procentriole formation (Habedanck et al., 2005). Following PLK4 localization, PLK4 is able to recruit, phosphorylate, and bind the protein STIL (Ohta et al., 2014). This leads to the recruitment of additional centriole components such as SAS6, CEP135, and γ‐tubulin, allowing for centriole duplication and assembly to begin (Kleylein‐Sohn et al., 2007; Ohta et al., 2014; Shyang Fong, Kim, Tony Yang, Liao, & Bryan Tsou, 2014). Centriole duplication produces two mitotic centrosomes, required for the formation of the microtubule‐based bipolar spindle during M‐phase. In the event of PLK4 overexpression or an S‐phase mitotic delay during this process, such as DNA damage, persistent PLK4 activity can cause centriole overduplication, or reduplication to occur (Habedanck et al., 2005). In contrast, when PLK4 is absent, either through knockdown or drug inhibition by centrinone, centriole duplication fails causing complete centriole loss after two or more consecutive cell cycles (Habedanck et al., 2005; Wong et al., 2015). This centriole loss eventually leads to p53‐dependent G1 cell cycle arrest. Together, this suggests that PLK4 has specific and timely roles in centriole duplication during S‐phase, and if the threshold expression of PLK4 is disrupted, either due to over‐ or under‐expression, centriole duplication is impacted.

PLK1 is also involved in centriole duplication, where its signaling allows for centriole disengagement, allowing for centriole duplication (Lon Carek, Hergert, & Khodjakov, 2010). Thus, a likely relationship exists between PLK1 and PLK4, where active‐PLK1 is required for centriole disengagement and maturation to occur during S‐phase, and PLK4 is required for duplication. The inherent nature of the relationship between PLK1 and PLK4, though, is not completely understood. Despite PLK4 being the major kinase responsible for centriole duplication, as described above, PLK1‐depletion has been implicated in causing a centriole duplication delay (Hatano et al., 2012; Lon Carek et al., 2010; Shukla, Kong, Sharma, Magidson, & Loncarek, 2015). It has been suggested that the reason for this delay is the inability of the cell to recruit centriole maturation factors, such as γ‐tubulin (Lon Carek et al., 2010). Alternatively, if PLK1 activity is elevated, the centrioles prematurely disengage, which allows the daughter centriole to recruit centriole proteins and nucleation factors, such as cenexin and γ‐tubulin, triggering an additional round of centriole duplication during S‐phase (Bryan Tsou et al., 2009; Kong et al., 2014; Lon Carek et al., 2010; Shukla et al., 2015). These studies support the idea that PLK1 and PLK4 are tightly regulated kinases with distinctly choreographed functions that work to ensure proper centriole disengagement, duplication, and recruitment of microtubule nucleating factors allowing for the assembly of a robust mitotic spindle.

## 4 FUNCTION OF PLK1

**Figure 3:**
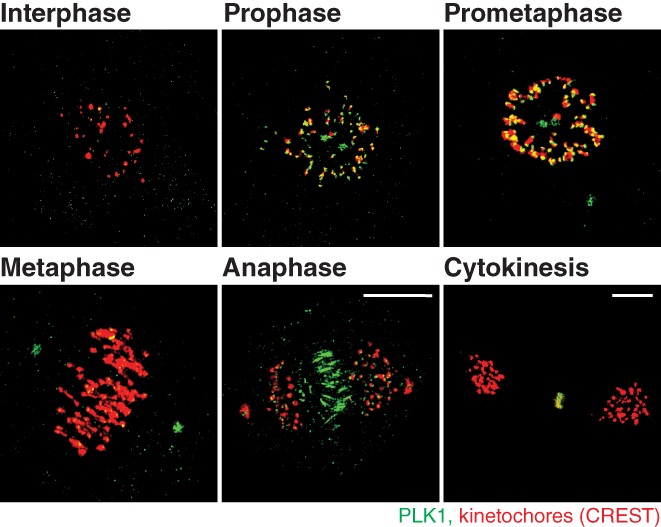
PLK1 distribution throughout mitosis. Structured illumination microscopy (SIM) volumetric projection micrographs of RPE cells showing localization of PLK1 (green) from interphase through cytokinesis. Kinetochore marker: CREST (red). Unpublished SIM micrographs from Dr. Heidi Hehnly's lab, performed by Erica Colicino

As shown in Figure 1, PLK1 contains two polo‐box regions, forming a PBD sufficient for localizing PLK1 to centrosomes, kinetochores and the cytokinetic midbody during mitosis (Figures 2 and 3). PLK1 spatial and temporal localization throughout division is shown using structured illumination microscopy (SIM) in Figure 3, illustrating its transition from centrosomes and kinetochores during early M‐phase to the midzone and cytokinetic midbody during the final stages of mitosis (Figure 3). By expressing the PLK1 kinase domain alone, it is insufficient to localize PLK1 at the mitotic centrosomes or kinetochores, whereas expressing the PBD alone localizes PLK1 robustly to mitotic centrosomes and, to a lesser extent, at kinetochores (Kishi, van Vugt, Okamoto, Hayashi, & Yaffe, 2009). Together, this suggested that even though the kinase domain was not required for PLK1 localization to mitotic centrosomes, it likely assists in PLK1 localization to kinetochores.

The PBD provides specific PLK1 binding through defined pS/pT sequences on binding scaffold proteins (Elia, Cantley, et al., 2003). This binding specificity requires PLK1 to contain both the two polo‐box regions within the PBD and the protein linker sequence separating the kinase domain from the PBD. To determine this PLK1‐specific binding sequence, a phospho‐peptide library was utilized. This library comprised of motifs known to be phosphorylated by alternative mitotic kinases, including CDKs and mitogen activated protein kinases (MAPKs). Using this library, the specific PLK1 PBD binding sequence was identified to be Ser‐pSer/pThr‐Pro/X (Elia, Rellos, et al., 2003). Once this sequence was determined, it was later used to identify greater than 600 potential putative PLK1 scaffolds to be further examined. This library of putative scaffolds are potential regulators of PLK1 that likely control PLK1’s spatial distribution and activity throughout the cell cycle (Lowery et al., 2007).

While only a select few PLK1 binding scaffolds have been confirmed, studies have clearly demonstrated that the subcellular distribution of PLK1 is tightly regulated through scaffold proteins. The regulation of these interactions relies heavily on the ability of these scaffolds to be phosphorylated by the mitotic kinase CDK1. The CDK1 kinase domain recognizes the motif Ser–Ser/Thr–Pro/X on PLK1‐binding scaffolds and is able to phosphorylate the second amino acid, known as priming, allowing for PLK1–PBD interactions (Enserink & Kolodner, 2010). Some of the identified scaffolds that require this priming by CDK1 are Bora, Gravin, and cenexin (Canton et al., 2012; Soung et al., 2009; Thomas et al., 2016). PLK1 also has the ability to phosphorylate its own scaffold, such as PBIP1 and BubR1 at the centromeres, a mechanism commonly used to maintain its localization at kinetochores (Elowe, Hümmer, Uldschmid, Li, & Nigg, 2007; Lee, Oh, Kang, & Park, 2008). These PLK1‐scaffold interactions allow for the localization and regulation of PLK1 dynamics, activity, and function at its subcellular locales.

Through differential regulation at numerous subcellular locales, PLK1 displays a variety of functions as a major regulator of mitotic entry. For instance, PLK1 localizes to the nucleus during G2‐phase where it has been implicated as a DNA damage checkpoint regulator (Hyun, Hwang, Hwan, & Jang, 2014; Smits et al., 2000; Van Vugt, Brá, & Medema, 2004; Wakida et al., 2017). During this process, PLK1 is required to recruit initial components of the DNA Damage Response (DDR), including ATM/ATR (Hyun, Hwang, et al., 2014). PLK1 is then dephosphorylated in an ATM‐Chk1 dependent manner, effectively inactivating the kinase. Thus, PLK1 is downregulated until the completion of the DDR, in which newly active‐PLK1 then works as a checkpoint regulator, allowing division to progress into M‐phase (Hyun, Hwan, & Jang, 2014; Lee, Hwang, & Jang, 2010). One possibility is that while DDR occurs, PLK1 is unable to recruit MT‐nucleating components to the centrosome so mitotic spindle formation cannot occur. An additional possibility that has yet to be examined is whether a population of PLK1 that acts within the nucleus can be exchanged to act on mitotic centrosomes. In this case, it is interesting to predict that PLK1 acts as a sensor that relays messages between the nucleus and mitotic centrosomes.

Since PLK1 depletion is lethal, it has been difficult to study its acute role at different cell‐cycle stages. For instance, PLK1 is thought to be necessary for cytokinesis, but it is difficult to test its direct contributions to this process because early mitotic defects caused by PLK1‐depletion triggers the spindle assembly checkpoint (SAC), preventing cells from entering cytokinesis and hindering these direct observations of PLK1 during this specific cell cycle stage. One solution has been to use small‐molecules that rapidly inhibit PLK1 during anaphase, after the SAC has been satisfied. ATP‐analogues that target and inhibit the PLK1 kinase domain in vitro include BI2536 and BI6727 (Lenart et al., 2007; Steegmaier, Hoffmann, Baum, Ter Lé Ná Rt, et al., 2007). However, the specificity of these drugs to specifically inhibit PLK1 in vivo is either poorly understood or unknown. In addition, based on the strong conservation of the PLK‐kinase domains, none of these compounds are expected to be selective for individual PLK‐homologues, complicating their use.

**Figure 4:**
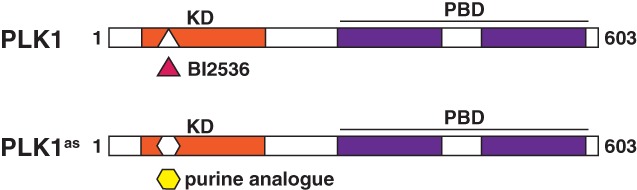
Model of PLK1 chemical genetics. The catalytic domain of PLK1 can be inhibited by treatment with an ATP analogue, such as the drug BI2536. By mutating and enlarging this catalytic domain, PLK1 can be inhibited in cells expressing this mutant using a purine analogue. Adapted from Burkard et al. (2007)

To overcome this issue, a chemical genetics system was developed in order to acutely inhibit specifically PLK1 at select times during division. A technique was developed based on traditional studies performed in yeast, where monospecific‐kinase inhibition could be achieved by replacing the target enzyme‐of‐interest with a variant, whose catalytic pocket was genetically modified to accept bulky purine analogues to inhibit kinase activity (Bishop et al., 2000). A similar approach can now be taken with mammalian tissue culture, where gene‐targeting and transgenic complementation can be used to establish somatic cell lines exclusively expressing an analogue‐sensitive PLK1 (PLK1^as^) (Figure 4; Burkard et al., 2007). These PLK1^as^ cells grow in culture similarly to wild‐type cells expressing endogenous PLK1. However, PLK1^as^ cells displayed heightened sensitivity to purine analogues, whereas wild‐type cells do not. PLK1^as^ cells, in the presence of purine analogues, display defects in mitotic spindle assembly, centrosome maturation, and chromosome alignment. These cells were then used to demonstrate and confirm PLK1’s involvement during cytokinesis, where treatment with a purine‐analogue during anaphase prevented cleavage furrow formation and abscission (Burkard et al., 2007). The system was also used to determine that PLK1 kinase functions that are separable by an activity threshold where titrating PLK1 activity leads to specific mitotic defects (Lera & Burkard, 2012). Together, these studies demonstrated the power of chemical genetics in dissecting complex, but short‐term, events in dividing cells.

While PLK1 is expressed at its peak level during metaphase (Golsteyn, Mundt, Fry, & Nigg, 1995), there are still small amounts of PLK1 at other cell cycle stages. For instance, at G1/G0 there is small but significant amount of PLK1 that may regulate cilia function (Wang et al., 2013). Such that PLK1 association with the BBsome, an octameric protein complex that localizes at the centrosome/basal body and is involved in trafficking cargoes to the primary cilium, is required for cilia disassembly as cells re‐enter the cell cycle. It is suggested that cilia disassembly can occur through two PLK1‐dependent pathways: (1) PLK1‐Dvl2 dependent AuroraA‐HEF1 recruitment to the ciliary base, leading to cilia disassembly through the noncanonical Wnt pathway (Lee et al., 2012) and (2) PLK1‐dependent phosphorylation DAZ‐interacting protein 1 (Dzip1), causing its dissociation from the BBsome and subsequent cilia disassembly (Zhang et al., 2016). Through the first pathway, PLK1 is recruited to cilia through Dvl2, a PLK1 scaffold that is primed for binding by Wnt5a. Once PLK1 binds Dvl2, it was shown that Aurora A and HEF1 expression increases, allowing a complex to form and be recruited to the cilia, leading to ciliary disassembly (Lee et al., 2012). In the secondary pathway, PLK1 phosphorylates Dzip1 at S210, allowing for its removal from the centriolar satellite, or ciliary base, triggering BBsome removal and eventual ciliary disassembly (Zhang et al., 2016). This suggests that while mitotic entry requires a robust amount of active‐PLK1 for mitotic fidelity, small amounts of precisely localized active‐PLK1 likely regulates essential, nonmitotic processes.

## 5 ROLE OF PLK1 AT CENTROSOMES, KINETOCHORES, AND CYTOKINETIC MIDBODIES

### 5.1 PLK1 at kinetochores

PLK1’s localization and function has been heavily studied at kinetochores, a complex of proteins associated with the centromere of a chromosome, where microtubules attach during cell division (reviewed in Saurin, 2018). PLK1‐localization at kinetochores is highest during prometaphase, where it is recruited initially by Bub1 (Qi et al., 2006), NudC (Nishino et al., 2006), and BubR1 (Elowe et al., 2007; Suijkerbuijk, Vleugel, Teixeira, & Kops, 2012). This localization is significantly reduced as the cell enters metaphase, similar to many other checkpoint signaling proteins, but PLK1 is not, in fact, required for spindle checkpoint function. PLK1 reduction at kinetochores results from SAC satisfaction during metaphase, where the CUL3‐based E3‐ligase ubiquitinates PLK1, resulting in its dissociation and the stabilization of kinetochore–microtubule attachments (reviewed in Liu & Zhang, 2017). This suggests that PLK1 activity suppresses kinetochore–microtubule dynamics. A potential mechanism by which PLK1 achieves this is through stabilizing initial microtubule attachments during prometaphase, such as through CLASP‐phosphorylation (Maia et al., 2012) and Kif2b (Hood, Kettenbach, Gerber, & Compton, 2012), while PLK1 removal during metaphase maintains dynamic microtubules (Liu et al., 2012). When there are no proper end‐on attachments at kinetochores, PLK1 can then act on BubR1 to recruit Mad2, preventing the passage of the SAC until proper end‐on attachments form (Wong & Fang, 2007). Once proper kinetochore–microtubule attachments are made, PLK1 activity decreases at kinetochores, allowing for passage of the SAC and initiation of the APC/Cyclosome, concluding metaphase (Beck et al., 2013). After cells enter anaphase/telophase, PLK1 is thought to transition from kinetochores into the midzone, where it recruits RhoGEF Ect2, allowing for proper cleavage furrow and cytokinetic bridge formation (Burkard et al., 2007; PLK1 at midzone shown in SIM micrograph in Figure 3).

In general, it is understood that increased PLK1 concentrations are found at kinetochores during prometaphase, but exactly how PLK1 operates within the kinetochore is uncertain. Specifically, it is unclear whether the spatial distribution of PLK1 within the kinetochore controls its accessibility to substrates, and its subsequent downstream functions, at this locale. However, the PLK1 spatial regulation at kinetochores remains enigmatic due to multiple PLK1 interactions and substrates along the kinetochore–centromere axis (Lera et al., 2016). For example, PLK1 interacts with outer kinetochore components, including Bub1, NudC, and BubR1, as well as inner kinetochore components where it is recruited by CENP‐U/50 (Kang et al., 2006) and CENP‐Q (Park et al., 2015). PLK1 also functions at the inner centromere, 500 nm from the outer kinetochore, through binding INCENP (Goto et al., 2006). A recent study utilized chemical genetics to investigate exactly how PLK1 spatial distribution within kinetochores contributes to its function and access to downstream substrates (Lera et al., 2016). This study was important in determining that pools of PLK1 anchored at one part of the kinetochore axis did not act on substrates localized at another point on the kinetochore axis. Additionally, the PLK1 pool that acts within the inner centromere is distinct in function from its role in stabilizing microtubule attachments at the outer kinetochore (Lera et al., 2016). This supported the idea that multiple pools of PLK1 exist at distinct kinetochore subcompartments, and that PLK1 displays discrete functions at these distinct sites.

### 5.2 *PLK1 at centrosomes*

PLK1 plays an essential role in the recruitment of MT‐nucleating components to mitotic centrosomes, an essential process for building a robust MT‐based spindle and ensuring mitotic fidelity. For instance, in *C. elegans*, PLK1 works to organize PCM scaffolding where its activity allows for the recruitment of PCM components SPD‐2 and SPD‐5. Strikingly, in an in vitro reconstitution environment, these two PCM components can self‐assemble into a selective phase PCM that is dependent on PLK1 activity (Woodruff et al., 2017). In vivo, PLK1 assists the assembly of pericentrin (Lee & Rhee, 2011) and CEP215 (Colicino et al., 2018; Santamaria et al., 2011) at the PCM through phosphorylation. Both pericentrin and CEP215 are essential PCM scaffolds required for the recruitment of additional PCM components, including γ‐turc. In the case of pericentrin, inhibiting its PLK1 phosphorylation sites, S1235 and S1241, results in the failed recruitment of PCM proteins, including γ‐tubulin, CEP192, and γ‐turc (Lee & Rhee, 2011). Little is known about CEP215’s phosphorylation by PLK1, except for an identified PLK1 phosphorylation site at S613 through a phosphoproteome (Santamaria et al., 2011). Our recent study dissected the role of the PLK1 scaffold Gravin and further explored the implications of CEP215 phosphorylation by PLK1 (Colicino et al., 2018). Interestingly, we found that when PLK1 is unable to be sequestered by Gravin at mitotic centrosomes, increased CEP215 phosphorylation occurred at its S613 site. Thus, we created a phospho‐mimetic mutant (CEP215‐S613E) to examine the downstream consequences of this phosphorylation. CEP215‐S613E expression led to CEP215 defocusing at mitotic centrosomes and caused chromosome mis‐segregation that further resulted in micronuclei formation (Colicino et al., 2018). Our study suggested that CEP215 phosphorylation status is required for mitotic fidelity, providing interesting implications as to when and where this phosphorylation should occur. One potential possibility is that CEP215 phosphorylation status is increased during metaphase exit, allowing the PCM to efficiently disassemble.

The centrosome proteins Gravin, CEP215, cenexin, pericentrin, primary microcephaly (MCHP2), and PLK1 have all been implicated in regulating the orientation of the mitotic spindle (Chen et al., 2014; Hanafusa et al., 2015; Hehnly et al., 2015; Hung, Hehnly, & Doxsey, 2016; Miyamoto et al., 2017), which can cause downstream consequences such as heart septation defects and microcephaly (Chen et al., 2014; Delaval & Doxsey, 2010; Vertii, Bright, Delaval, Hehnly, & Doxsey, 2015). However, it is unknown how all these molecules act in concert to correctly position the spindle along the division axis. We do know that if we un‐couple PLK1 from its scaffold Gravin, increased phosphorylation of its downstream substrate CEP215 occurs, which is associated with a loss of astral microtubules and corresponding spindle positioning defects (Colicino et al., 2018; Hehnly et al., 2015). It is unknown, though, where the mother centriole appendage protein cenexin, or the PCM proteins pericentrin and MCPH2 fits into this possible pathway. In order to better understand the role of PLK1 at the mitotic centrosomes, the chemical genetics system described above could be used to replace endogenous‐PLK1 at a single locus with either a kinetochore‐targeted or centrosome‐targeted PLK1^as^ (PLK1‐PACT used in Kishi et al., 2009). This will allow centrosome‐ or kinetochore‐tethered PLK1 to be inhibited using a purine analogue or wild‐type PLK1 using a common PLK1 inhibitor (BI 2536) in order to tease out the consequences of PLK1 at the mitotic centrosomes versus the kinetochores. In addition, just as with the kinetochore (Lera et al., 2016), PLK1^as^ could be tethered to subcompartments of the centrosome, where it is known to have a specific function (e.g., PCM, centriole, or mother centriole appendages) (Colicino et al., 2018; Hehnly et al., 2015; Lee & Rhee, 2011; Soung et al., 2009). These studies could elucidate whether PLK1 operates in pools within the centrosome, as it seems to operate at the kinetochore (Lera et al., 2016).

### 5.3 *PLK1 at the midbody*

PLK1 activity is required for cytokinesis and is regulated, in part, through binding phosphorylated scaffold proteins with distinct subcellular localization. During metaphase, CDK1 predominately creates the phosphorylated docking sites for PLK1, but what controls PLK1 docking post‐anaphase and during cytokinesis when less CDK1 activity is present? Similar to kinetochore‐localized PLK1 scaffolds, the microtubule‐associated protein regulating cytokinesis (PRC1) is phosphorylated by PLK1, creating a PLK1 docking site on PRC1. Interestingly, PRC1 is phosphorylated by CDK1 adjacent to this PLK1 docking site during metaphase to prevent PLK1 binding at this time. PLK1 binding to PRC1 is necessary though for cytokinesis (Hu et al., 2012; Neef et al., 2007), where it phosphorylates MgcRacGAP/Cyk4 on several residues and elicits the binding of epithelial cell transforming sequence 2 (Ect2) (Burkard, Maciejowski, Rodriguez‐Bravo, Repka, & Lowery, 2009; Burkard et al., 2007; Wolfe, Takaki, Petronczki, & Glotzer, 2009), a guanine nucleotide exchange factor for the small GTPase RhoA (Somers & Saint, 2003). MgcRacGAP phosphorylation by PLK1 is crucial to trigger the onset of cytokinesis.

Following the completion of the cytokinetic furrow, many of the central spindle components are packaged into a structure known as the cytokinetic midbody. The midbody lies within the intercellular bridge, which connects the two daughter cells. The abscission of the intercellular bridge occurs in the vicinity of the midbody, where numerous abscission proteins are enriched, including PLK1 (reviewed in Chen, Hehnly, & Doxsey, 2012). Thus, the midbody is a likely platform to coordinate abscission machinery where kinases act to ensure faithful abscission. One example of this is with the midbody localized protein CEP55. CEP55 is first phosphorylated by PLK1 where it prevents CEP55’s association with the midzone (Bastos & Barr, 2010). Only after the loss of PLK1 activity does CEP55 translocate to and integrate into the midbody. Inhibition of PLK1 causes CEP55 to prematurely translocate to the midbody, causing abscission failure. The likely reason for this failure is that aberrant midbody architecture arises and the inability to target ESCRT‐III components to the midbody, such as ALIX and TSG101 (Kamranvar et al., 2016; Morita et al., 2007). Thus, PLK1 seems to regulate cytokinesis progression and faithful abscission through its ability to recruit midbody components in an orderly manner by phosphorylation of substrates.

Three‐dimensional structured illumination microscopy (SIM) and electron microscopy tomography were used to examine fluorescently tagged or immunostained components of ESCRT‐III [e.g., CHMP4B (charged MVB protein 4B) and VPS4B (vacuolar protein sorting‐associated protein 4B)]. This work showed that ESCRT‐III concentrates initially at the midbody and then at a separate site in the intercellular bridge (Elia, Sougrat, Spurlin, Hurley, & Lippincott‐Schwartz, 2011; Guizetti et al., 2011), where an array of helical filaments are assembled (Agromayor et al., 2009; Guizetti et al., 2011; Yang et al., 2008). The ESCRT‐III‐dependent helical filaments allow for deformation of the intercellular bridge membrane adjacent to the midbody, allowing the bridge to sever. Recent studies have implicated PLK1‐dependent phosphorylation of a proline‐rich domain of ALIX is required to transform ALIX from a closed conformation to an open conformation, allowing it to function in cytokinetic abscission (Sun et al., 2016). This suggests a major mechanism for PLK1 in activating ESCRT function of ALIX to induce abscission.

## 6 PLK1‐SCAFFOLD INTERACTIONS

**Figure 5:**
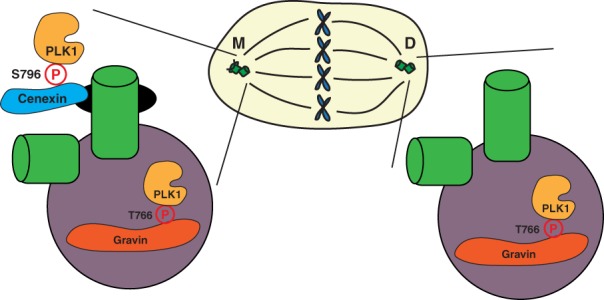
PLK1 scaffolding at the mitotic centrosomes. A model depicting the localization of PLK1 (gold) to the mother centriole appendages (black) and pericentriolar matrix (PCM, purple) through its binding scaffolds, cenexin (blue) and Gravin (red). PLK1 has been shown to bind cenexin, a known mother centriole appendage protein, at its phosphorylated S796 site (Soung et al., 2009). PLK1 has also been shown to bind Gravin, a known PCM component, at its phosphorylated T766 site (Canton et al., 2012). These scaffolds subsequently sequester PLK1 at its subcentrosomal locales, regulating its activity during mitosis

While the function of PLK1 at various locales is intriguing and necessary to understanding its importance during division, it is equally as important to understand how PLK1 is subcellularly localized and regulated. The proteins required to ensure this tightly choreographed regulation and localization are known as binding scaffolds. The binding of PLK1 to its scaffold can assist in regulating PLK1’s activity in three possible ways: (1) To augment or enhance its ability to phosphorylate downstream substrates; (2) To insulate or sequester it to a specific locale; (3) To terminate or impede its ability to phosphorylate downstream substrates (Langeberg & Scott, 2015). While there is much known about PLK1 and the mitotic scaffold Bora (Bruinsma et al., 2016; Macůrek et al., 2008; Seki et al., 2008; Thomas et al., 2016), here, we will focus on what is known about PLK1 scaffolds specifically at mitotic centrosomes and our recent study involving PLK1 and its PCM localized scaffold Gravin (Figure 5). Strikingly, the two mitotic centrosomes, which appear to be symmetric in nature, are actually asymmetric structures (Nigg & Stearns, 2011). While there are PLK1 scaffolds within the PCM (Canton et al., 2012; Colicino et al., 2018; Hehnly et al., 2015), a presumably symmetric structure between the two mitotic centrosomes, there is also an identified scaffold that localizes exclusively to one of the two mitotic centrosomes (Figure 5, (Soung et al., 2006). This creates an additional layer of regulation for the amount and activity of PLK1 that can occur between the two centrosomes and suggests interesting roles for PLK1 in regulating spindle orientation and cell differentiation.

The asymmetry between the two mitotic centrosomes derives from the centriole duplication cycle, where one centriole is always inherently older than the other. The oldest centriole, referred to as the mother, is structurally distinct from the youngest centriole, or daughter (Nigg & Stearns, 2011). The mother centriole contains unique proteins that make up the distal and subdistal appendages, one of which has been identified as a PLK1‐scaffold protein, cenexin. PLK1 directly binds cenexin at its S796 site after CDK1 phosphorylation (depicted in Figure 5; Soung et al., 2009). This interaction has been implicated in regulating the recruitment of the PCM proteins γ‐tubulin and pericentrin to mitotic centrosomes during division. When this interaction is disrupted through mutating the PLK1 binding site (S796A), these components fail to be recruited (Soung et al., 2009). This is interesting to put into context of another study that found a unique set of mother‐centriole appendage proteins known to require cenexin for appendage localization that include centriolin and ninein that interact with pericentrin to regulate spindle orientation (Chen et al., 2014). Cenexin regulates both distal and subdistal appendage formation; however, it is unknown whether this process requires cenexin's interaction with PLK1. Additional studies have shown that cenexin is vital to orient the mitotic spindle parallel to the plane of 3D‐epithelial expansion (Hung et al., 2016) and mediate the propensity for stem cells to mis‐segregate chromosomes towards the mother mitotic centrosome (Gasic, Nerurkar, & Meraldi, 2015). However, it is not clearly understood whether the interaction between cenexin and PLK1 is required for these processes. Together, it is interesting to design a testable model where cenexin phosphorylation during mitotic entry recruits a complex involving pericentrin and CEP215 to the oldest mitotic centrosome to help direct spindle orientation through modulation of the nucleating capacity of the oldest mitotic centrosome.

**Figure 6:**
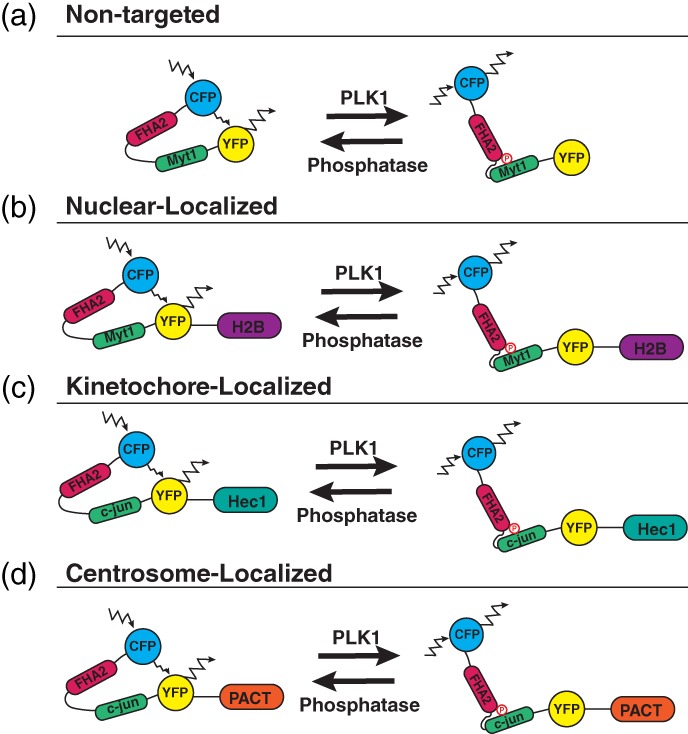
PLK1 FRET biosensors. (a) A nontargeted PLK1 FRET biosensor containing a CFP monomer (blue), FHA2 phospho‐binding domain (magenta), Myt1 PLK1‐substrate sequence (green), and YFP monomer (yellow). When active‐PLK1 is present, it phosphorylates the Myt1 sequence (T495), causing a conformational change in the biosensor, decreasing FRET. When no active‐PLK1 is present, the biosensor is in a relaxed state, allowing for FRET (Macůrek et al., 2008). (b) A nuclear‐localized PLK1 FRET‐biosensor fused to H2B (purple) (Bruinsma, Raaijmakers, & Medema, 2012). **(c)** A kinetochore‐localized PLK1 FRET‐biosensor containing a c‐jun PLK1‐susbtrate sequence and fused to Hec1 (cyan). The c‐jun substrate sequence was mutated (S17T), allowing for PLK1‐specific phosphorylation of the biosensor (Liu et al., 2012). **(d)** A centrosome‐localized PLK1 FRET‐biosensor containing a c‐jun PLK1‐substrate sequence and fused to the pericentrin AKAP centrosomal‐targeting (PACT) domain (orange) (Colicino et al., 2018)

Another PLK1 scaffold that localizes to mitotic centrosomes, specifically within the PCM, is Gravin (Figure 5). Gravin is a scaffold protein that interacts with numerous kinases, including protein kinase A (PKA), protein kinase C (PKC), Aurora A, and PLK1 at various cell cycle stages. Gravin is specifically phosphorylated at T766 by CDK1, allowing for the direct binding of PLK1 during cell division (depicted in Figure 5, Canton et al., 2012). Gravin sequesters Aurora A and PLK1, facilitating a kinase phosphorylation cascade where Aurora A phosphorylates PLK1 at its T210 site, which subsequently increases PLK1 activity (Hehnly et al., 2015). Our recent studies additionally demonstrated that when Gravin is lost in advanced stage prostate cancers, there is an increased incidence in PLK1‐associated errors, including mitotic delay and chromosome instability (Colicino et al., 2018). While these studies provided an understanding that Gravin forms a complex with PLK1 at mitotic centrosomes during mitosis, it was unclear what significance this interaction had on PLK1 spatial and temporal dynamics and subsequent activity. To test this, we utilized a stable GFP‐PLK1 cell line along with Fluorescent Recovery After Photobleaching (FRAP) to calculate that Gravin anchors approximately 12% of PLK1 at mitotic centrosomes at metaphase. We next utilized a Fluorescence Resonance Energy Transfer (FRET) biosensor for PLK1 activity (Macůrek et al., 2008) and anchored it to the mitotic centrosomes in order to measure PLK1 activity specifically at mitotic centrosomes (Figure 6). Using this biosensor, we could calculate that when Gravin is removed from cells, there is a significant increase in active‐PLK1 at mitotic centrosomes. This suggested that when PLK1 is anchored by Gravin, it is unable to act on its downstream substrates. Thus, when Gravin is lost, increased PLK1‐dependent phosphorylation of its downstream centrosome substrate CEP215 (Santamaria et al., 2011) is measured, resulting in the defocusing and disorganization of CEP215 at mitotic centrosomes (Colicino et al., 2018). The downstream consequences of this disorganization includes the loss of centrosome function through decreased microtubule re‐nucleation, loss of stable microtubules, and increased incidence of genomic instability measured by micronuclei formation (Colicino et al., 2018). While these studies provide insight as to how PLK1 is regulated at the mitotic centrosomes during division, it is still unclear how additional PLK1 scaffolds regulate PLK1 and how PLK1 is regulated between the two mitotic centrosomes.

### 6.1 Innovative tools developed to study PLK1 spatial and temporal dynamics, activity, and function during division

The development of a PLK1‐FRET biosensor allowed, for the first time, the activity of PLK1 to be examined in an in vivo setting throughout the cell cycle (Bruinsma et al., 2016; Macůrek et al., 2008). By manipulating a previously developed Aurora B kinase activity biosensor (Fuller et al., 2008), a biosensor was developed that possessed a donor (CFP) and acceptor (YFP), a phospho‐binding domain (FHA2, Forkhead‐Associated Domain 2), and a PLK1 specific phosphorylation sequence (Myt1). When little or no PLK1 activity is present, the biosensor is in a relaxed state, where the excitation and emission of CFP causes energy transfer which causes the excitation and emission of YFP, or FRET (refer to Figure 6a). When PLK1 is present and active, it is able to phosphorylate the Myt1 substrate sequence. This causes the FHA2 domain to bind, leading to a conformational change in the biosensor, so the excitation and emission of CFP no longer causes the excitation/emission of YFP, or a loss of FRET. By taking a ratio of FRET vs no FRET, the user of this biosensor can arbitrarily measure the temporal activity of PLK1 within the cell. It was improved even further by changing PLK1 substrate sequence to the more specific c‐jun, and further increasing PLK1 specificity by mutating the PLK1‐phosphorylation site from serine to threonine (Figure 6, Liu et al., 2012). While this biosensor was revolutionary in measuring and visualizing PLK1 activity in vivo throughout cell division, it was cytosolically expressed and only provided PLK1 temporal activity. From there, scientists wanted to be able to additionally examine the spatial activity of PLK1 during division.

This biosensor was then targeted to specific locales to examine PLK1 activity at unique sites during the cell cycle such as the nucleus (Figure 6a,b, Bruinsma et al., 2015; Macůrek et al., 2008), kinetochores (Figure 6c, Liu et al., 2012), and then the centrosome (Figure 6d, Colicino et al., 2018). PLK1 activation was first identified to occur several hours before mitotic entry, where it requires Aurora A‐dependent phosphorylation of Thr210 in the T‐loop of the PLK1 kinase domain (Macůrek et al., 2008). This was identified using a novel PLK1 FRET‐biosensor which localized within the nucleus and cytosol (Figure 6a, Macůrek et al., 2008). This study also determined that Aurora A‐dependent activation of PLK1 at Thr210 was enhanced when the scaffold Bora was present. A follow‐up study used a modified FRET‐biosensor fused to histone‐2b (H2B), identifying that PLK1 activity is increased within the nucleus during G2 in a Bora‐dependent manner (Figure 6b, Bruinsma et al., 2015). Interestingly, a recent study identified a mechanism for nuclear localization of PLK1 where PBD binding to the kinase domain masks a nuclear localization signal in PLK1. Phosphorylation of the kinase domain within the T‐loop leads to exposure of an NLS causing the entry of PLK1 into the nucleus during G2 (Kachaner et al., 2017).

The PLK1 FRET‐biosensor has also been anchored to kinetochores by fusing to the kinetochore protein Hec1 (Figure 6c, Liu et al., 2012). These studies designed a FRET‐based phosphorylation sensor to track phosphorylation changes at the kinetochores during division in live cells. This sensor demonstrated that when PLK1 levels were high on kinetochores, such as when cells are treated with the microtubule‐depolymerizing drug nocodazole, that phosphorylation of the kinetochore‐targeted biosensor was high. When PLK1 concentration on kinetochores was low, such as during metaphase when chromosomes are aligned along the metaphase plate, phosphorylation of the biosensor was low compared to nocodazole‐treated cells. Based on this consistency, where the PLK1 activity biosensor correlated with relative concentrations of PLK1 levels at kinetochores, the biosensor was used to track phosphorylation dynamics as chromosomes align during metaphase. These studies demonstrated that as the kinetochores aligned at metaphase, the kinetochore‐anchored PLK1 biosensor was dephosphorylated in a phosphatase 1 (PP1)‐dependent manner. In addition, they found that phosphatase levels are inversely correlated with PLK1 recruitment (Liu et al., 2012). This is consistent with a phosphorylation‐dependent mechanism to regulate PLK1 localization, likely through PBD binding to phosphorylated kinetochore scaffolds (Elia, Rellos, et al., 2003). Thus, PLK1‐biosensors not only can be utilized to understand PLK1‐activity, but also the significance of PLK1‐substrate dephosphorylation (Liu et al., 2012).

To examine PLK1‐activity specifically at centrosomes, the FRET‐biosensor was fused to the pericentrin AKAP450 centrosomal‐targeting domain (PACT), allowing for specific localization to centrosomes (Figure 6d, Colicino et al., 2018). Our study utilized PLK1‐FRET‐PACT to understand how a PLK1 scaffold, in this case Gravin, could spatially coordinate PLK1 activity at centrosomes during metaphase (Colicino et al., 2018). These studies identified that when one specific centrosome‐localized scaffold was depleted, there was an increase in PLK1 substrate phosphorylation at the centrosome that correlates with an increase in centrosome disorganization and loss of nucleation potential during prometaphase (Colicino et al., 2018). Together, these studies shed light on the tight regulation of PLK1 at mitotic centrosomes and how this works to ensure proper mitotic centrosome formation and function. With further development of these technologies, it is hopeful that the interactions between PLK1 at its subcellular locales can be thoroughly examined, determining how and when PLK1 responds to cellular cues in an effort to ensure mitotic fidelity.

### 6.2 PLK1 as a targeted cancer therapeutic

Another major field in PLK1 research is developing PLK1 small‐molecule inhibitors as drug therapies in diseases such as cancer (reviewed in Elizabeth, Gutteridge, Ndiaye, Liu, & Ahmad, 2016; Murugan et al., 2011). Numerous studies have tested PLK1 inhibitors, including BI2536, as potential cancer therapeutics for advanced metastatic tumors, including prostate cancer (Hou et al., 2013; Zhang et al., 2014), lung cancer (Awad et al., 2017; Breitenbuecher et al., 2017), neuroblastoma (Pajtler et al., 2017), and non‐Hodgkin's lymphoma (Vose et al., 2013) to name a few. These studies suggest elevated levels of PLK1 expression in highly metastatic and advanced cancers, leads to chromosome instability and aneuploidy (Yamamoto et al., 2006), providing these cells an advantage to overgrow and invade tissues. By inhibiting PLK1 activity in these cells, it is hypothesized that the cells will suffer cell cycle arrest, leading to cell death and hindered tumor growth, allowing for increased survival (Raab et al., 2015). Initial studies using 2D tissue culture and mouse models of various cancers yielded promising results. The inhibitor BI2536 impeded tumor growth in a mouse xenograph models (Steegmaier, Hoffmann, Baum, Lenart, et al., 2007). The overall survival of the animals significantly increased compared to controls, suggesting PLK1 inhibitors would be promising cancer therapeutics in clinical trials.

Despite these results, clinical trials for PLK1 therapeutics have not yet been successful as monotherapy treatments, but appear to work best in clinical trials within a chemotherapeutic cocktail (Yim, 2013). One reason for this is that PLK1 inhibition causes sensitivity to other pathway inhibitors, such as androgen signaling inhibitors, preventing prostate cancer tumor growth (Zhang et al., 2014). An alternative method for targeting PLK1 activity in these cells is to better understand how PLK1 is regulated in cells, specifically through binding scaffolds as described above. The PLK1 scaffold Gravin is commonly misregulated in advanced prostate cancer and its depletion has been shown to increase PLK1 activity (Canton et al., 2012; Colicino et al., 2018). If these kinase‐scaffold interactions can be disrupted instead of the kinase itself, there is the potential to increase the efficacy of PLK1 drug therapeutics.

### 6.3 Current gaps of knowledge in research

Despite the new and innovated tools and technologies available, there is still a lot to learn about PLK1 regulation and function at its various subcellular locales during division. For instance, while a few newer studies have emerged looking at PLK1 during cytokinesis and abscission, it is still unclear whether PLK1 is active and functional during abscission and whether there is a direct role for PLK1 to ensure proper cleavage of the cytokinetic bridge. It has been suggested that PLK1 works as a negative‐feedback and checkpoint regulator at this final stage of division, ensuring that ESCRT‐complex components are properly recruited, and the cell is prepared to undergo abscission. Despite these studies, a lot more work needs to be done in order to measure PLK1 dynamics and activity at these sites, to determine specific PLK1 binding scaffolds and downstream phosphorylation targets, and to test whether PLK1 has a direct role in ensuring abscission. The development of a midbody‐localized PLK1 FRET biosensor would provide an opportunity to further study the spatial and temporal activity of PLK1 at the cytokinetic midbody. From here, PLK1’s interactions with CEP55 and its role in cytokinesis and abscission can be more clearly understood. While some of the tools are available and predictions have been made on a number of candidates for binding scaffolds and phosphorylation targets, many of these candidates have not been confirmed. By utilizing a combination of innovative tools, including chemical genetics and FRET‐biosensors, with the addition of super resolution microscopy, it is possible to determine PLK1’s direct, and indirect, roles in ensuring successful division at mitotic centrosomes, kinetochores, and the cytokinetic midbody.
